# Prognostic Impact of An Integrative Landscape of Clinical, Immune, and Molecular Features in Non-Metastatic Rectal Cancer

**DOI:** 10.3389/fonc.2021.801880

**Published:** 2022-01-07

**Authors:** Soledad Iseas, Juan M. Sendoya, Juan Robbio, Mariana Coraglio, Mirta Kujaruk, Vanesa Mikolaitis, Mariana Rizzolo, Ana Cabanne, Gonzalo Ruiz, Rubén Salanova, Ubaldo Gualdrini, Guillermo Méndez, Marina Antelo, Marcela Carballido, Cecilia Rotondaro, Julieta Viglino, Martín Eleta, Alejandro Di Sibio, Osvaldo L. Podhajcer, Enrique Roca, Andrea S. Llera, Mariano Golubicki, Martín Carlos Abba

**Affiliations:** ^1^ Oncology Unit, Gastroenterology Hospital “Dr. Carlos Bonorino Udaondo”, Buenos Aires, Argentina; ^2^ Laboratorio de Terapia Molecular y Celular, Genocan, Fundación Instituto Leloir, IIBBA (CONICET), Buenos Aires, Argentina; ^3^ Unidad de Investigación Traslacional, Laboratorio de Biología Molecular GENUIT, Gastroenterology Hospital “Dr. Carlos Bonorino Udaondo”, Buenos Aires, Argentina; ^4^ Proctology Unit, Gastroenterology Hospital “Dr. Carlos Bonorino Udaondo”, Buenos Aires, Argentina; ^5^ Pathology Unit, Gastroenterology Hospital “Dr. Carlos Bonorino Udaondo”, Buenos Aires, Argentina; ^6^ Biomakers Molecular Pathology and Research, Buenos Aires, Argentina; ^7^ Imaxe Image Diagnosis Center, Buenos Aires, Argentina; ^8^ Hospital General de Agudos “Dr.Cosme Argerich”, Buenos Aires, Argentina; ^9^ Basic and Applied Immunological Research Center, School of Medical Sciences, National University of La Plata, La Plata, Argentina

**Keywords:** rectal cancer, non-metastatic, mutational profile, biomarkers, precision medicine

## Abstract

Rectal Cancer (RC) is a complex disease that involves highly variable treatment responses. Currently, there is a lack of reliable markers beyond TNM to deliver a personalized treatment in a cancer setting where the goal is a curative treatment. Here, we performed an integrated characterization of the predictive and prognostic role of clinical features, mismatch-repair deficiency markers, HER2, CDX2, PD-L1 expression, and CD3^−^CD8^+^ tumor-infiltrating lymphocytes (TILs) coupled with targeted DNA sequencing of 76 non-metastatic RC patients assigned to total mesorectal excision upfront (TME; n = 15) or neoadjuvant chemo-radiotherapy treatment (nCRT; n = 61) followed by TME. Eighty-two percent of RC cases displayed mutations affecting cancer driver genes such as *TP53*, *APC*, *KRAS*, *ATM*, and *PIK3CA*. Good response to nCRT treatment was observed in approximately 40% of the RC cases, and poor pathological tumor regression was significantly associated with worse disease-free survival (DFS, HR = 3.45; 95%CI = 1.14–10.4; p = 0.028). High neutrophils-platelets score (NPS) (OR = 10.52; 95%CI=1.34–82.6; p = 0.025) and *KRAS* mutated cases (OR = 5.49; 95%CI = 1.06–28.4; p = 0.042) were identified as independent predictive factors of poor response to nCRT treatment in a multivariate analysis. Furthermore, a Cox proportional-hazard model showed that the *KRAS* mutational status was an independent prognostic factor associated with higher risk of local recurrence (HR = 9.68; 95%CI = 1.01–93.2; p <0.05) and shorter DFS (HR = 2.55; 95%CI = 1.05–6.21; p <0.05), while high CEA serum levels were associated with poor DFS (HR = 2.63; 95%CI = 1.01–6.85; p <0.05). Integrated clinical and molecular-based unsupervised analysis allowed us to identify two RC prognostic groups (cluster 1 and cluster 2) associated with disease-specific OS (HR = 20.64; 95%CI = 2.63–162.2; p <0.0001), metastasis-free survival (HR = 3.67; 95%CI = 1.22–11; p = 0.012), local recurrence-free survival (HR = 3.34; 95%CI = 0.96–11.6; p = 0.043) and worse DFS (HR = 2.68; 95%CI = 1.18–6.06; p = 0.012). The worst prognosis cluster 2 was enriched by stage III high-risk clinical tumors, poor responders to nCRT, with low TILs density and high frequency of *KRAS* and *TP53* mutated cases compared with the best prognosis cluster 1 (p <0.05). Overall, this study provides a comprehensive and integrated characterization of non-metastatic RC cases as a new insight to deliver a personalized therapeutic approach.

## Introduction

Colorectal cancer is the third most common cancer worldwide, accounting for approximately 10% of solid tumors (1). Rectal cancer (RC) comprises 40% of all colorectal cancers, with about 70–75% staged as a non-metastatic disease at the initial diagnosis. The RC incidence and mortality are expected to increase substantially by 2035 (2, 3). The clinical management of RC is mainly dependent on tumor staging at diagnosis (4), and total mesorectal excision (TME) is considered the cornerstone of curative treatment for early-stage tumors. Since the preoperative chemoradiotherapy (CRT) followed by TME was established as the standard strategy for locally advanced rectal cancer (LARC), the local recurrence rate was reduced approximately 5% (5, 6). More recently, the development of the total neoadjuvant therapy (TNT) whereby consolidation chemotherapy is given after chemoradiotherapy for LARC treatment (7–9) has resulted in an increased probability of complete pathological response (pCR), improved tumor resectability, and sphincter preservation without compromising local tumor control (10, 11). However, the current 5-year survival rate remains approximately 65% (12).

Multiple studies indicate that tumor response to preoperative treatment strongly predicts the disease-free survival of patients (13–16). There is a spectrum of tumoral response to TNT in which up to 20–30% of patients will have a pCR heralding an excellent prognosis (17). In contrast, up to 40% of patients will not respond, resulting in minimal to no regression or disease progression, even during CRT. It is not currently possible to predict which patients will have a favorable response to therapy, and such heterogeneous responses can finally impact long-term oncological outcomes (18, 19). Identifying good and poor responders before neoadjuvant treatment may help clinicians consider more personalized strategies that include intensive preoperative treatment, such as TNT and upfront surgery to prevent unnecessary treatment-related toxicities and non-operative management (20). It is also essential to balance the risk of local and metastatic recurrence to avoid over-treatment and preserve organ function and life quality (20).

Several clinicopathological and molecular features has been associated with a prognostic and/or predictive value in RC such us the mucinous histology (21, 22), the unresponsiveness associated with mismatch repair-deficient tumors (23), loss of CDX2 expression (24), elevated pretreatment CEA levels (25), high serum inflammation markers (26, 27), and the association between a low CD3 and CD8 tumor-infiltrating lymphocytes density in the pretreatment biopsies and minimal regression to CRT (28). Tumor tissue-based molecular predictors of response to nCRT in LARC patients have been extensively studied. Several studies observed that *KRAS* mutation and combined *KRAS*/*TP53* mutations are associated with resistance to CRT and poor oncological outcomes (29–31). Moreover, several promising gene expression signature-based classifiers have been reported (32). However, there is no consensus regarding the role of these prognostic and predictive factors, probably because they derived from retrospective studies not independently validated in prospective external cohorts (32). In this context, no reliable prognostic and predictive biomarkers have been identified beyond the TNM, and there is no consensus regarding the role and implementation of the molecular-based biomarkers. Tumor heterogeneity undoubtedly also plays a relevant role in determining a diverse spectrum of treatment responses and oncological outcomes that need to be considered in biomarker discovery strategies (33).

This study aimed to characterize a prospective, single center-based cohort of non-metastatic rectal cancer staged by MRI at the clinical, immunological, and molecular levels. It aims towards identifying predictive and prognostic factors capable of guiding treatment selection and stratifying patients under curative approaches.

## Materials and Methods

### Rectal Cancer Cohort

This prospective translational study comprised 76 consecutive eligible non-metastatic rectal cancer patients recruited and treated at the Oncology Unit at the Gastroenterology Hospital “Dr. Carlos Bonorino Udaondo” (Buenos Aires, Argentina) between November 2015 and September 2018. Inclusion criteria were: i) available pre-treatment formalin-fixed paraffin-embedded (FFPE) biopsy, ii) histologically confirmed adenocarcinoma, and iii) absence of distant metastases at baseline. Initial clinical staging was based on rectoscopy, thorax–abdomen computed tomography (CT) scan, and pelvic magnetic resonance imaging (MRI). Clinical data collected from patient medical records included age at diagnosis, gender, distance to anal verge, risks factors according to ESMO rectal cancer guidelines (4), CEA and CA19.9 values, histological features, mismatch repair (MMR) protein status by immunohistochemistry, and neutrophil-platelet score (NPS). All patients gave their informed consent for inclusion before they participated in the study. The study was conducted in accordance with the Declaration of Helsinki, and the protocol was approved by both the Udaondo Hospital Ethics Committee and the Fundación Instituto Leloir Ethics Committee.

### Treatment and Follow-Up

All cases were discussed in our multidisciplinary team (MDT). Those patients without locally advanced disease were assigned to TME surgery upfront (n = 15/76). Our standard routine approach for delivery of neoadjuvant therapy for LARC as the initial therapeutic approach define intermediate/locally advanced rectal cancer as very low cT2-T3ab, cT3cd-T4, extramural vascular invasion (EMVI) positivity, high mesorectal nodes burden or mesorectal nodes unlikely amenable for quality TME, circumferential radial margin (CRM) involvement, and lateral lymph node dissemination (LLND). All LARC patients (n = 61/76) were assigned to standard pelvic long course radiotherapy (LCRT: 50.4 Gy in 28 fractions of three-dimensional conformal radiotherapy, 1.8 Gy per fraction, per day) with concurrent capecitabine (825 mg/m^2^/bid for 28 days), termed hereafter CRT. Patients with a high risk of systemic relapse (EMVI, high mesorectal node burden and LLND) underwent induction chemotherapy (I + CRT), which comprises pre-treatment before the CRT with three cycles of CAPOX (130 mg/m^2^ of oxaliplatin on day 1 and capecitabine 1,000 mg/m^2^/bid, days 1–14 every 3 weeks). Two cycles of capecitabine monotherapy (850 mg/m^2^/bid, days 1–14 every 3 weeks) was then administered until response assessment for all patients. Together, I + CRT and CRT are referred to as nCRT throughout this manuscript.

Response assessment was measured within 6–8 weeks of completing radiotherapy by digital rectal examination (DRE), CT, and MRI (ymrTN and mrTRG) (34). Pathological tumor regression (pTRG) was evaluated on the surgical specimen using the Protocol for the Examination of Specimens from Patients with Primary Carcinoma of the Colon and Rectum v.4.0.1.0 recommended by the College of American Pathologists (CAP) (35). Response to nCRT was also evaluated using the NAR score (36). Patients with low rectal tumors and clinical complete response (cCR) by DRE and diffusion-weighted MRI (DW-MRI) (ymrT0N0, mrTRG = 1, low/lack of signal in DW-MRI) were exempted from surgery and were followed up every three months for the first two years and every six months thereafter. The remaining patients underwent a TME 12 to 16 weeks after completing radiotherapy. Adjuvant treatment was considered for patients with postoperative residual tumor presence associated with histopathological high-risk factors. Results shown in this paper include follow-up for progression/relapse and survival status until August 2020.

### Sample Collection and Quality Assessment

All sample collection procedures were carried out according to institutional Standard Operating Procedures for frozen and FFPE specimens based on international consortia recommendations. Baseline tumor biopsies were collected as part of the rectoscopy diagnostic procedure and were divided into two blocks: one block underwent snap-freezing with liquid nitrogen and the other was prepared as FFPE. The latter was analyzed for the presence of at least 60% adenocarcinoma with hematoxylin/eosin staining. The snap-frozen mirror biospecimen was processed for molecular studies, while the FFPE was stored for immunohistochemical studies. Cold ischemia times were strictly monitored and registered in order not to exceed 30 min from extraction to fixation (formalin or freezing). On the day of collection of the diagnosis tissue sample, peripheral blood samples were also collected according to Standard Operating Procedures.

### Immunohistochemistry Analysis (IHC)

The immunohistochemistry (IHC) tests were performed using the automatized platform Bond-Max Leica Biosystems for the antibodies: MLH1 (clone G168-728; Cell Marque), MSH2 (G219-1129; Cell Marque), MSH66 (PU29; Cell Marque), PMS2 (NOR4G; Cell Marque). Tumors were considered negative for MLH1, MSH2, PMS2, or MSH6 expression only if there was a complete absence of nuclear staining in the tumor cell, while positive expression was defined as the presence of nuclear staining of tumor cells, irrespective of the proportion or intensity. Infiltrating lymphocytes, stromal cells and adjacent non neoplastic epithelium served as internal positive controls. Immunostaining was performed using a Roche Benchmark XT system and anti-CD3 (Clone 2GV6, Ventana—Roche), anti-CD8 (Clone SP57, Ventana—Roche), anti-HER2/Neu (Clone 4B5, Ventana—Roche), and anti-PD-L1 (Clone SP263, Ventana—Roche) antibodies. Immunostaining was evaluated by two independent qualified pathologists. In four cases of discrepancy, an additional assessment was performed by a third senior pathologist. For CD3 and CD8, average values were obtained from examining all intra and peritumoral areas. A semi-quantitative score was defined; CD3 and CD8 expression was classified according to the percentage of total tumor-related lymphocyte (peritumoral and intratumorally) staining: low (0–34%), moderate (35–64%), and high (65–100%). PD-L1 was evaluated using the Combined Positive Score (CPS) established for gastric/gastroesophageal junction adenocarcinoma (37). HER-2/Neu scoring was performed according to the College of American Pathologists (CAP), which describes three categories: HER2-negative (0; 1+), HER2-equivocal (2+) and HER2-positive (3+) (38). Complete absence for CDX2 with positive internal controls was considered negative, while any percentage of tumor at any intensity of staining was considered positive. Immunostaining for CDX2 was performed using the Leica Bond system (Clone EPR2764Y, Cell Marque) (39).

### DNA Purification and Targeted DNA Sequencing

DNA was extracted from FFPE primary tumor biopsies using a QIAamp DNA FFPE Tissue Kit (Qiagen, Hilden, Germany). DNA quality was evaluated based on the absorbance ratios of A260/280 and A260/230 using a NanoDropTM 2000c Spectrophotometer (Thermo Fisher, MA, USA). DNA quantity was determined using the Qubit^®^ 2.0 Fluorometer with the Qubit^®^ dsDNA HS Assay Kit (Thermo Fisher). Two independent Targeted DNA sequencing panels were employed to allow the mutational profiling of 72 cancer driver genes (see **Supplementary Data 1**). DNA libraries that were built with GeneRead DNAseq Colorectal Cancer Panel V2 were processed and analyzed as was previously described (31). Of note, DNA libraries that were constructed with the AmpliSeqTM for Illumina Cancer Hotspot Panel v2 Kit that allow the detection of 2,800 COSMIC mutations from 50 oncogenes and tumor suppressor genes, were prepared with 100 ng of genomic DNA as was previously described (40). These DNA libraries were measured using Qubit^®^ 2.0 Fluorometer with the Qubit^®^ dsDNA HS Assay Kit (Thermo Fisher). All libraries were above the minimum concentration requirement of 2 nM for further sequencing in an Illumina MiSeq platform.

### Bioinformatic Analysis

Quality control of sequencing data was performed in all samples using the Real-Time Analysis software sequence pipeline from Illumina. The short-read sequences were aligned against the human reference genome (Build Hs37d5, based on NCBI GRCh37) using the Burrows–Wheeler aligner (BWA-MEM) algorithm. Subsequent mutational analysis was performed at mean coverage depth ≥200 reads. Variants were filtered out when the alternative allele depth was lower than 10 reads. The GATK Mutect2 toolkit (https://gatk.broadinstitute.org/) was used for single nucleotide variant (SNV) calling. Variant annotation was performed using several resources and databases such as: SnpEff, dbNSFP, PhyloP, SIFT, PolyPhen2, MutationTaster, LRT, and CADD. The GnomAD resource (https://gnomad.broadinstitute.org/) was used to evaluate variant frequency in the global population. All mutations were evaluated using the Integrative Genomics Viewer (https://software.broadinstitute.org/software/igv/). To perform a comparative analysis of the mutational profile identified in our cohort of patients (HBU), we analyzed rectal cancer datasets obtained from The Cancer Genome Atlas (TCGA) and the Memorial Sloan Kettering Cancer Center project (MSKCC) retrieved from the cBioPortal resource (http://www.cbioportal.org/). The DNA sequencing data can be found at SRA (ID: PRJNA633284) and **Supplementary Data 1**.

### Statistical Analysis

To compare categorical data between groups, the Chi-square test or Fisher’s exact test were used. Continuous data were compared with the Student’s t-test or Wilcoxon rank sum test. Survival curves were estimated using the Kaplan–Meier method and compared using the log-rank test. Two-tailed p-values were calculated and p-values <0.05 were considered as significant. The primary endpoints of clinical interest were CRT response evaluated by CAP, local and distal recurrence risk (local recurrence-free survival and metastasis-free survival), and DFS and OS as secondary endpoints. DFS was defined as the time from the first day of CRT until clinical or radiological disease recurrence or death from any cause. OS was defined as the time from treatment initiation to death from any cause. We used the cox proportional hazards regression models to estimate hazard ratios (HR) and 95% confidence intervals (CI) for the associations between the factors and follow-up in a step-by-step approach. First, univariable logistic regression was performed on variables of interest in relation to the outcome. Second, only variables detected in more than 90% of the analyzed cases which achieved p <0.1 in the univariable analysis were included for further evaluation in a multivariable Cox proportional hazards model. In our final multivariate model only statistically significant associated variable (p <0.05) were included adjusted for the following covariates: pT, pN, pCRM, NPS, CEA, CA19.9, perineural invasion, and age. We noticed no violation of the proportional hazards assumption in visual inspection of log–log plots and Schoenfeld residuals plotted against follow-up time. The hierarchical clustering on principal components (HCPC) method provided by the FactoMineR R TME upfront surgery package (http://factominer.free.fr/) was employed to identify patient clusters in an unsupervised and multivariate approach. Briefly, the Principal Component method is used as a preprocessing step for the clustering in order to denoise the data and to balance groups of variables included in the model. The PCA representation is also used to visualize the hierarchical tree and/or the partitions before the hierarchical clustering of patients based on Euclidean distances. The included clinicopathological and molecular variables were: treatment (CRT, I + CRT, and TME upfront surgery), gender, age (<50 years old), distance, cT3/T4, pT3/T4, cN, pN, EMVI, cCRM, pCRM, lateral lymph node dissection, CEA, CA19.9, NPS, NAR, CAP, Downstaging, adjuvancy, TME, diseases progression (local recurrence, metastasis or death), TILs density, perineural invasion, vascular invasion, MMR, *KRAS*, *APC*, and *TP53* mutational status. Cluster characterization was performed by visual representation of the v-test values associated with variables that were significantly contributing with the clusters partition (p <0.05). All statistical data analyses were performed using R Statistical Software. This study complied with reporting recommendations for tumor marker prognostic studies (REMARK) criteria.

## Results and Discussion

### Patient Cohort and Treatment Response

**Figure 1:**
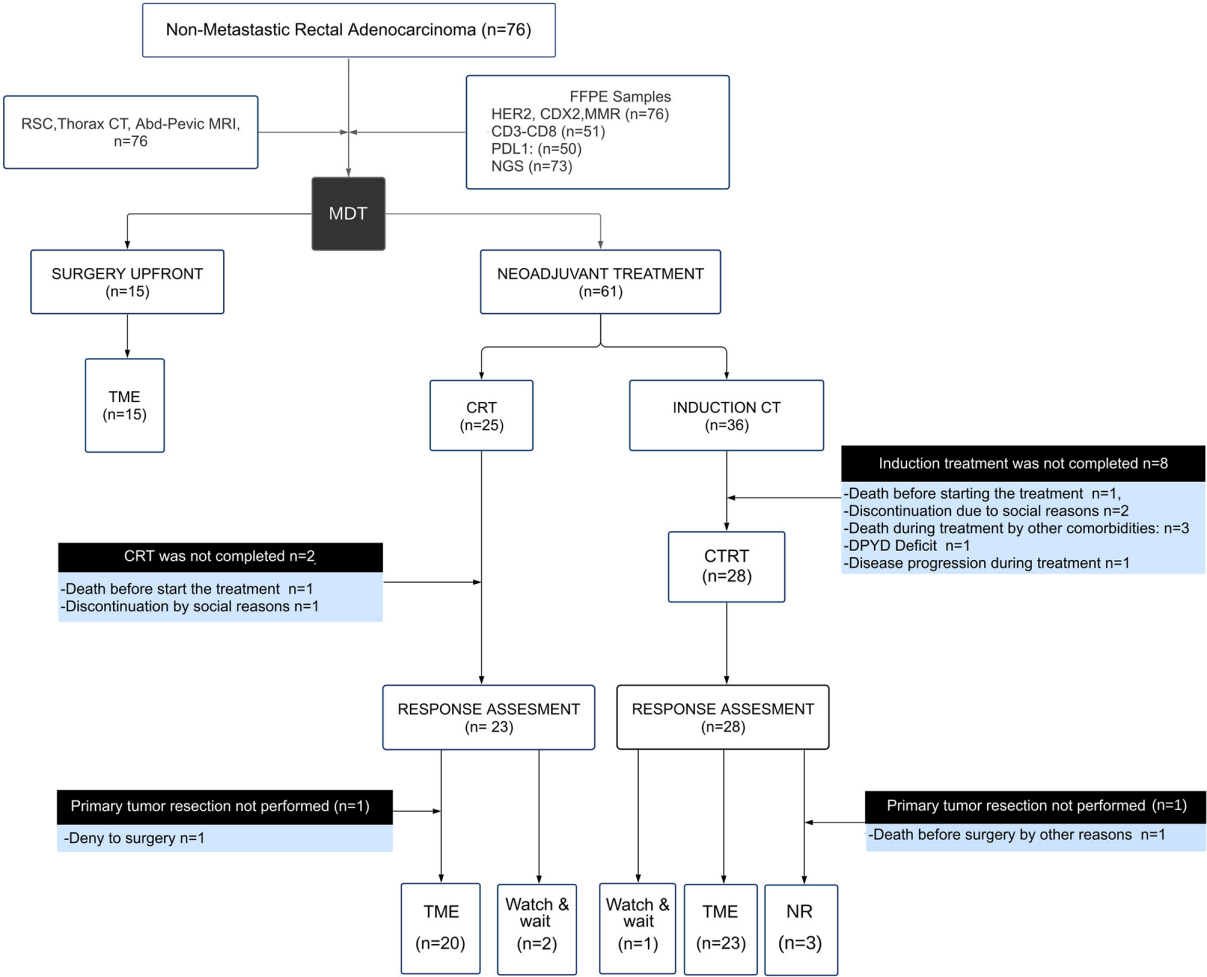
Description of the study design and participants recruited by the multidisciplinary team (MDT). Flow chart showing the composition of the cohort, outcomes and results. Response assessment to neoadjuvant treatment (CRT) was determined in 49 patients as indicated at the bottom of the flow chart, including 43 total mesorectal excisions (TME), 3 watch and wait, and 3 non-resectable (NR) cases.

Seventy-six non-metastatic RC patients were enrolled for the present study (**Figure 1**). The median age at diagnosis was 61 years old, outlining that 28% (20 out of 76) of patients were under 50 years at diagnosis. Sixty-one locally advanced rectal cancer patients (61 out of 76) were assigned to CRT neoadjuvant therapy (25 to CRT and 36 to I + CRT) followed by TME surgery (**Table 1**). The remaining fifteen patients (15 out of 76) without locally advanced disease were assigned to TME upfront surgery (**Table 1**). Extended clinicopathological, demographic, and molecular data are summarized in **Supplementary Table 1**. The demographic characteristics of this cohort are in agreement with previously reported ones, confirming the high prevalence of males (67%) and young patients, which is coincident with a sustained increased incidence of CRC in young people worldwide, particularly in RC (41–44). The cohort was characterized by intermediate and high-risk tumors at diagnosis: cT3–cT4 (85.5% of the cases), stage III (50%), EMVI+ (33%) and MRC+ (71%), similarly to previous studies in our country (16, 45). Mucinous adenocarcinomas were observed in 10% of the cases, with nearly all locally advanced disease at diagnosis and a significant association with treatment assignment (p = 0.04; **Supplementary Table 1**).

**Table 1:** Clinical and demographics data of the non-metastatic rectal cancer cohort according to the treatment assigned.

Patient Characteristics* (n = 76)	CRT (n = 25)	I + CRT (n = 36)	Upfront surgery (n = 15)
Median age at diagnosis	63 (54–69)	59 (45–64)	64 (51–68)
Gender			
Female	8 (32)	11 (31)	6 (40)
Male	17 (68)	25 (69)	9 (60)
Distance from the anal verge			
0–70 mm	11 (44)	17 (47)	4 (27)
71–120 mm	9 (36)	15 (42)	9 (60)
>120 mm	5 (20)	4 (11)	2 (13)
TNM**^#^**			
Stage I (T1–T2, N0)	1 (4)	0 (0)	10 (67)
Stage II (T3–T4, N0)	18 (72)	5 (14)	4 (27)
Stage III (any T, N+)	6 (24)	31 (86)	1 (6)
EMVI^#^			
Positive	6 (24)	18 (50)	1 (7)
Negative	19 (76)	18 (50)	14 (93)
CRM**^#^**			
Positive	18 (72)	34 (94)	2 (13)
Negative	7 (28)	2 (6)	13 (87)
Lateral lymph nodes**^#^**			
Present	1 (4)	12 (33)	0 (0)
Absent	24 (96)	24 (67)	15 (100)
Histology**^#^**			
Mucinous	0 (0)	7 (19)	1 (7)
Others	25 (100)	29 (81)	14 (93)

^*^Number of patients (%) unless otherwise stated.

^#^Statistically significant differences among treatments (p <0.05).

With respect to the subgroup of patients who underwent direct surgery (n = 15), a total mesorectum excision (TME) was performed in all patients. The median number of nodes resected was 14 (range 9–28). Complete mesorectum plane and negative margins were obtained in all subjects. Oxaliplatin-based adjuvant therapy was administered in 7 out of 15 cases due to the presence of involved lymph nodes.

The response to neoadjuvant treatment was evaluated in 49 patients 8 weeks after completing the CRT treatment as described in **Figure 1**. A limiting factor of the current study behind the sample size was the discontinuation of the neoadjuvant treatment in 20% of the patients (12 out of 61) due to comorbidities and socioeconomic factors. The median number of resected lymph nodes was 13 (range 8–24). Complete tumor regression (adding cCR and pCR) was 18% in agreement with previous prospective studies and institutional series (14, 46–48). The EMVI and CRM negativization after neoadjuvant treatment was 87.5 and 77.5%, respectively. A good response to neoadjuvant treatment (defined as cCR or CAP G0–G1) was achieved by 41% of the patients (20 out of 49) (**Supplementary Table 2**). On the other hand, a poor response to neoadjuvant treatment (defined as CAP G2–G3 and/or unresectable tumors) was presented by 59% of the patients (29 out of 49). According to NAR score assessment, 37.8% of the patients showed a score below 8, and downstaging was observed in 57% of the cases (28 out of 49). When CRT response was evaluated by CAP, NAR, and pathological downstaging, we observed a good correlation between parameters. However, when CAP, NAR, and pathological downstaging were evaluated with the appearance of long-term oncological events, only the CAP showed a significant association (p <0.05).

Seventy-six RC patients were evaluated for the initial descriptive analysis, of which 12 were excluded due to insufficient follow-up. Thus, the entire cohort that completed the planned treatment with follow-up data was 64 patients (**Figure 1**). The median follow-up time was 22.5 months (IQR 7–34 months) after TME surgery of which 6 patients presented local recurrence (8%), 4 presented synchronous local and distant progression (6%), and 14 developed only distant metastases (22%). The predominant metastatic pattern was at the liver, lungs and with less frequency on retroperitoneal lymph nodes and peritoneum. During follow up, 13 patients died (20%), of which 11 were due to disease (17%). The estimates for 2-year DFS and OS were 65 and 80%, respectively. Usually, the highest risk of local and distant recurrence in rectal cancer is presented during the first two years of surveillance, which coincides with the median follow-up of our cohort.

### Analysis of Mismatch Repair Protein Deficiency and Immune-Related Markers

Several genomic and epigenomic studies have contributed to the understanding of the molecular pathogenesis of CRC, allowing the classification of patients in the microsatellite stable (MSS) and the microsatellite instability-high (MSI-H) groups. The MSS group constitutes 85% of all CRCs cases and exhibits proficient DNA mismatch repair mechanisms (pMMR), and low tumor mutational burden (TMB) (49–51). While the MSI-H group constitutes the remaining 15% of the cases and is characterized by defects in the DNA mismatch repair program (dMMR), frequently resulting in a high TMB. The microsatellite stability status of all non-metastatic RC was evaluated by IHC expression analysis of the mismatch repair proteins MLH1, MSH2, MSH6, and PMS2 for their further classification in pMMR or dMMR tumors. We detected 7% of dMMR cases (5 out of 76) of which four were patients assigned to the I + CRT treatment (p >0.05). Rectal location has been identified as the tumor location with the lowest prevalence of tumors associated with dMMR (2–5%) that is in agreement with our results (52, 53). Interestingly, a gradient from dMMR to pMMR has been described in the right colon (22.3%), left colon (4.6%), and rectum (0.7%) (52). The median age of the dMMR patients was 49 years and their associated MSI-H tumors were characterized as MSH2-MSH6 deficient and not cases of MLH1 deficiency were detected. Regarding the clinical presentation, 4 out of 5 dMMR patients required neoadjuvant treatment based on induction followed by CRT due to locally advanced disease. These cases were reported as poor responders to neoadjuvant therapy. The lower tumor regression rate efficacy of dMMR cases and even tumor progression with neoadjuvant regimens based on oxaliplatin has recently been described in prospective and retrospective trials (23, 54). While 84% of the young RC patients (21 out of 25) were characterized as pMMR tumors with locally advanced disease at diagnosis that have been previously associated with patients with delayed diagnosis (55, 56). Previous studies have showed that dMMR CRC patients are significantly more sensitive to immune checkpoint inhibitors than pMMR cases (57, 58). Furthermore, a recent study revealed that dMMR CRC are related to an pro-inflammatory tumor microenvironment, increased expression of immune-related genes and enhanced immunogenicity compared to pMMR cases (59).

**Figure 2:**
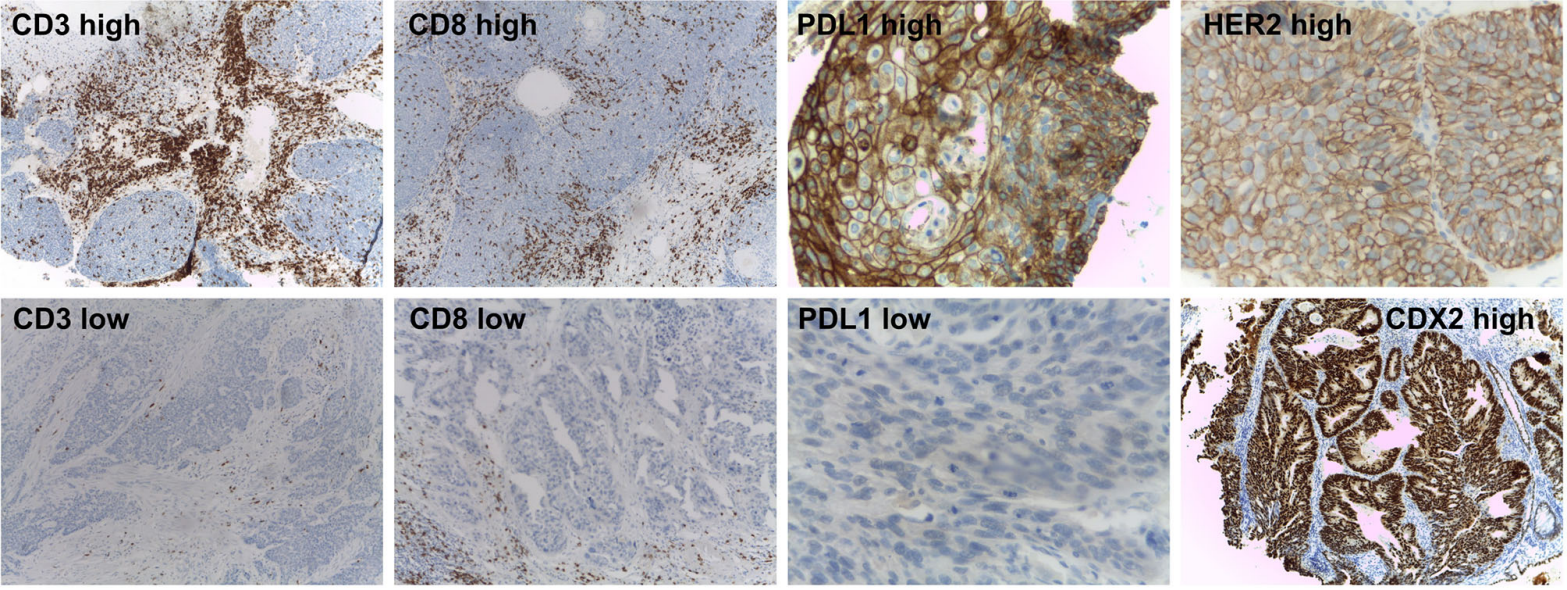
Immunohistochemical markers assessed in the non-metastatic rectal cancer cohort. Representative immunohistochemistry results for high and low CD3 and CD8 TILs, PD-L1 expression (up and down panels respectively), high HER2 and CDX2-expressing tumors.

CD3 and CD8 tumor-infiltrating lymphocytes (TILs) and PD-L1 expression were evaluated in fifty-one RC patients that underwent neoadjuvant therapy (**Figure 2**). CD3 and CD8 TILs density was low in 75% (38 out of 51) and 92% (47 out of 51) of RC respectively. While moderate CD3 (13 out of 51) and CD8 (2 out of 51) TILs density were detected in the remaining cases. PD-L1 positivity (CPS >1%) was detected in 20% of RC patients that underwent neoadjuvant therapy (10 out of 51), and high PD-L1 expression levels (CPS >10%) was detected in only one of the positive cases (1 out of 10). While the remaining 80% of RC samples showed low PD-L1 expression levels (CP S<1%) (41 out of 51). The low CD3^−^CD8 TILs density and PD-L1 expression detected are in agreement with a previous transcriptomic-based study reported by us that classified the non-metastatic RC as CMS2 (31), that are mainly associated with a poorly immunogenic stromal component (60). Finally, when HER2 and CDX2 immunodetections were assessed, two cases of HER2 expression were detected (2 out of 76), while CDX2 was expressed in almost all non-metastatic RC cases (73 out of 76). The low prevalence of CDX2 negative (4%) and HER2 positive (3%) cases is coincident with previously reported series (24, 61, 62). No statistically significant associations were found between HER2, CDX2, CD3^−^CD8 TILs density, and PD-L1 expression and CRT treatment response and outcomes (p >0.05) (**Supplementary Table 1**).

### Mutational Profile and Predictive and Prognostic Factors

**Figure 3:**
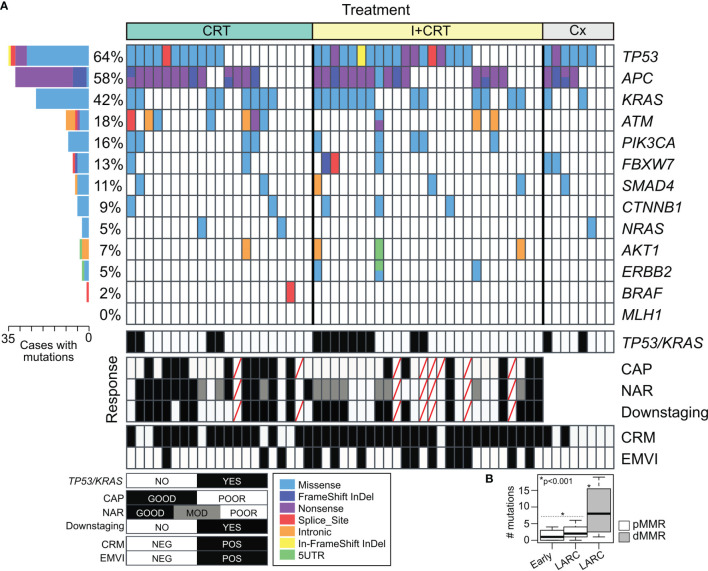
Mutational profile of non-metastatic rectal cancer based on two Targeted DNA Sequencing panels. **(A)** Tile plot showing recurrent altered cancer driver genes in RC cases according to the treatment assigned and response to the preoperative neoadjuvant treatments. **(B)** Box plot of the number of mutations in early-stage tumors and locally advanced rectal cancer (LARC) among proficient (pMMR in white) and deficient mismatch repair (pMMR in gray) rectal cancer.

**Figure 4:**
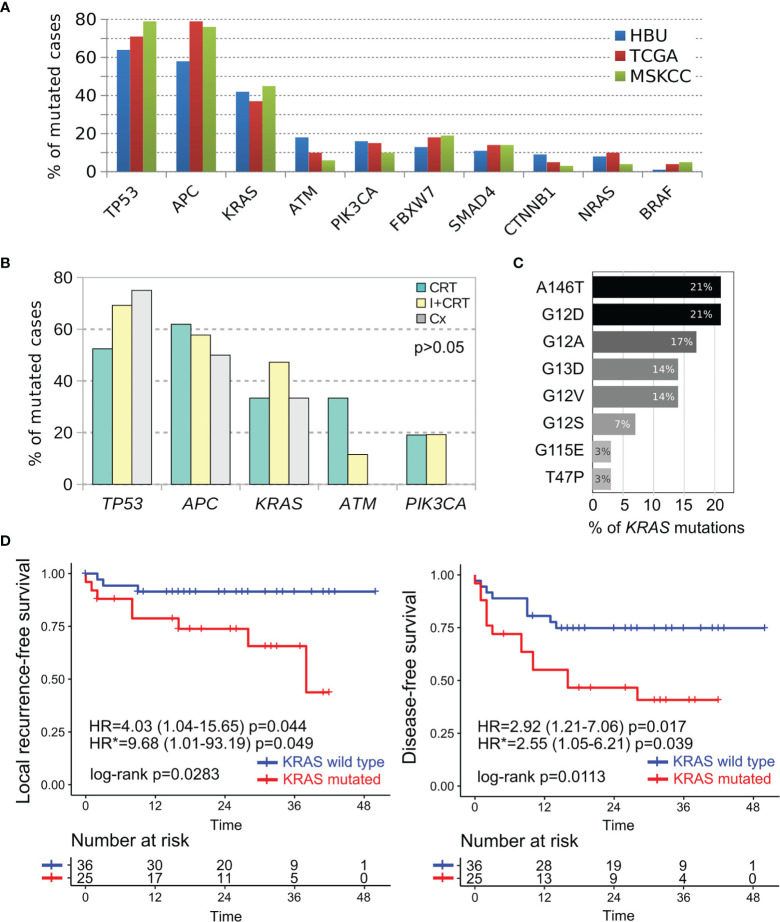
Comparative mutational profile of the most prevalent cancer driver genes in non-metastatic RC cases. **(A)** Comparative frequency of mutations in our non-metastatic cohort (HBU in blue bars), TCGA (red bars) and MSKCC (green bars) RC cohorts. **(B)** Comparative analysis of the most frequently mutated cancer driver genes according treatment assignment (CRT in green bars, I + CRT in yellows bars and TME upfront surgery in gray bars). **(C)** Most frequently *KRAS* missense mutations detected among RC cases. **(D)** Univariate Kaplan–Meier survival analysis and Cox regression analysis according the *KRAS* mutational status of RC cases. Survival analysis revealed that the KRAS mutated cases were particularly associated with shorter local recurrence-free survival and disease-free survival as showed by their hazard ratios determined in the univariate (HR) and multivariate (HR*) models.

Targeted DNA sequencing was performed in 55 pretreatment biopsies using the Illumina Cancer Hotspot Panel and the GeneRead DNAseq Colorectal Cancer Panel in 18 and 37 RC cases respectively. Furthermore, *KRAS*, *NRAS*, and *BRAF* mutational status were complementary obtained by direct PCR sequencing in 18 additional RC patients, totalizing 73 out of 76 included cases. We detected 230 somatic mutations among 82% of RC cases (60 out of 73) including 54% missense mutations (123/230), 20% nonsense mutations (46/230), 14% intronic variants (33/230), 6% Indel frameshift mutations (13/230), 4% splice site mutations (10/230), 1% In-frame deletions (3/230), and others (**Supplementary Data 1**). Among the most frequently mutated genes, we detected *TP53* (64%), *APC* (58%), *KRAS* (42%), *ATM* (18%), and *PIK3CA* (16%) (**Figure 3**). The comparative frequency of mutations of the non-metastatic RC cases of our cohort (HBU) and the derived from the TCGA and MSKCC datasets is shown in **Figure 4A**. Similar mutational frequency distributions were observed across cohorts, where the most frequently mutated genes were *TP53*, *APC*, *KRAS*, *ATM*, and *PIK3CA*. The rectal carcinomas from patients assigned to CRT were characterized by an increased frequency of mutated cases (90% for CRT and 92% for I + CRT) compared with the TME upfront surgery group (75%) (p <0.05). However, no significant associations with response to treatment were observed (p >0.05) (**Figure 4B**). In addition, patients with dMMR consistently presented the highest rates of mutations detected in the cohort compared with pMMR LARCs and early-stage RC cases (p <0.001) as expected of a hypermutator phenotype (**Figure 3B**) (63–65).

The majority of the alterations found in the *TP53* gene were the ‘hotspot’ mutations involving R175H, R248Q, and R273C/H positions. These codons are among the most frequently mutated in CRC patients and lead to the loss of the DNA-binding capability and the TP53 transcriptional activity function. APC gene was predominantly characterized by stop gain mutation in 81% of the mutated cases (26 out of 32) followed by InDel frameshift mutations (**Figure 3A**). Tumors harboring *KRAS* alterations were characterized by the predominant presence of the A146T, G12D/A/V/S activating mutations followed by G13D, T74P, and G115E AA substitutions (**Figure 4C**). *KRAS* mutation is the most common canonical gain-of-function mutation in CRC, and earlier functional studies clearly demonstrated that mutant *KRAS* leads to an epidermal growth factor receptor-independent disturbance of the RAS/RAF/MAPK pathway, which regulates cell proliferation and survival in CRC (66, 67). We were also able to detect potentially actionable mutations in *PIK3CA* involving C420R, E542K, Q546K, and H1047R positions, although they do not have sufficient evidence to be included in treatment guidelines.

When clinicopathological and molecular features were evaluated in univariate and multivariate models to determine their independent predictive values, high NPS (OR = 10.52 95%CI = 1.34–82.64; p = 0.025) and *KRAS* mutated cases (OR = 5.49; 95%CI = 1.06–28.40; p = 0.042) were associated with poor response to neoadjuvant treatment (**Supplementary Table 3**). In addition, a poor pathological tumor regression (CAP 2–3) showed a statistically significant association with worse outcome (HR = 3.45 95%CI = 1.14–10.44; p = 0.028). Patients with CAP 0–1 showed an estimated DFS at 50 months of 80% vs. 40% in those with CAP 2–3 (p = 0.0175).

To further evaluate the independent prognostic value of the clinicopathological and molecular features, we next performed a multivariate Cox proportional-hazard analysis that included relevant prognostic factors such as: pT, pN, pCRM, NPS, CEA, CA19.9, perineural invasion, and age. This analysis showed that the *KRAS* mutational status was independently associated with higher risk of local recurrence (HR = 9.68; 95%CI = 1.01–93.2; p <0.05) and shorter DFS (HR = 2.55; 95%CI = 1.05–6.21; p <0.05) (**Figure 4D**); while high CEA serum levels were associated with worse DFS (HR = 2.63; 95%CI = 1.01–6.85; p <0.05) (**Supplementary Figure 1**). Furthermore, increased CEA levels and *KRAS* mutated cases were also associated to worse metastasis-free survival and disease-free survival in univariate analysis (**Supplementary Figure 1**). Regarding cancer specific OS, *KRAS* mutated, pCRM and pT3-T4 were associated to higher mortality rates due to cancer (**Supplementary Figure 1**). Overall, the results show that the *KRAS* mutational status was highly informative as independent prognostic and predictive marker in non-metastatic RC patients adding relevant information beyond that provided by the standard clinical factors.

### Integrative and Unsupervised Analysis of RC Patients

**Figure 5:**
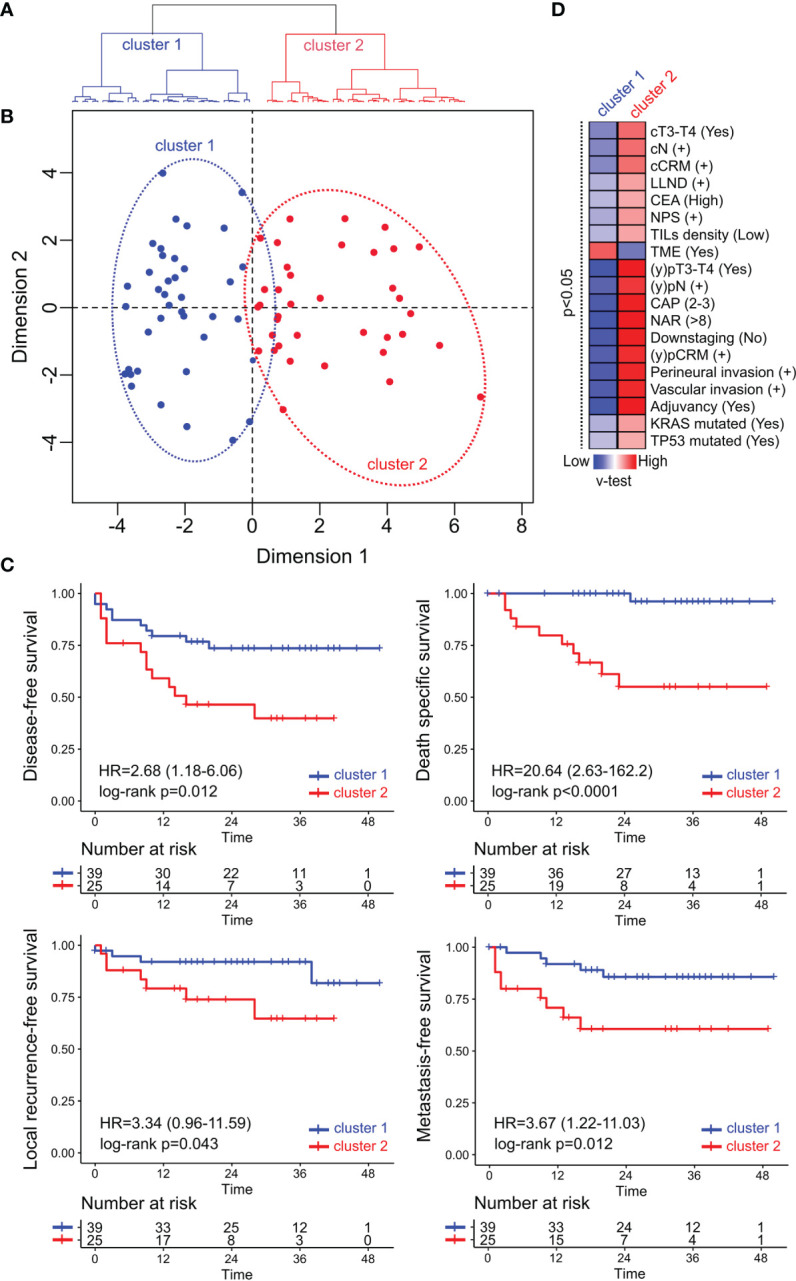
Multivariate and unsupervised analysis of clinicopathological, immune and mutational markers of the non-metastatic RC cohort. **(A)** The seventy-six patients were segregated into two classes: cluster 1 (in blue) and cluster 2 (in red) based on the first bifurcation of the dendrogram produced by the hierarchical clustering partitioning analysis of samples. **(B)** Multidimensional scaling plot showing the euclidean distance of each sample from each other determined by their similarities in the included variables. **(C)** Univariate Kaplan–Meier survival analysis RC cases according to their assigned cluster. Survival analysis revealed that cluster 2 was particularly associated with shorter disease-free survival, death-specific survival, local recurrence-free survival, and metastasis-free survival compared with cluster 1. **(D)** Heatmap of the significant statistical variables (p <0.05) that contributes with clusters discrimination based on positive (in red) and negative (in blue) v-test values.

Hierarchical Clustering on Principal Components (HCPC) method was applied with the aim to identify cluster of non-metastatic RC patients with shared clinicopathological and molecular features. Unsupervised analysis demonstrates a clear segregation of RC samples in two distinctive clusters based on the first bifurcation of the clustering dendrogram (**Figure 5A**) or in the similarity distances from dimension 1 in the multidimensional scaling plot (**Figure 5B**) based on the 28 integrated variables. The RC cluster 1 was constituted by 39 patients of which 69% were assigned to CRT/I + CRT (27 out of 39) and 31% to upfront surgery (12 out of 39). While the RC cluster 2 was composed by 37 patients of which 92% (34 out of 37) were assigned to CRT/I + CRT and 8% to upfront surgery (3 out of 37). Univariate survival analysis revealed that the RC cluster 2 was particularly associated with shorter overall specific survival (HR = 20.64; 95%CI = 2.63–162.2; p <0.0001), metastasis-free survival (HR = 3.67; 95%CI = 1.22–11.03; p = 0.012); local recurrence-free survival (HR = 3.34; 95%CI = 0.96–11.59; p = 0.043) and disease-free survival (HR = 2.68; 95%CI = 1.18–6.06; p = 0.012) compared with the good prognosis cluster 1 (**Figure 5C**). The multivariate Cox proportional-hazard analysis including the clinicopathological factors used to identify the RC clusters showed a non-independent association between variables as expected. We then identified the statistically significant variables contributing to the clusters partition using a v-test based on the hypergeometric distribution to characterize the patients’ composition of the non-metastatic RC clusters (**Figure 5D**). The worst prognosis cluster 2 was enriched by stage III/IV, NAR >8, CAP 3–4, pCRM+, high NPS cases with vascular and perineural invasion. The best prognosis cluster 1 was characterized by moderate clinical risk tumors and good responders to nCRT (**Figure 5C**). Furthermore, the worst prognosis cluster 2 was also enriched with 53% of *KRAS* mutated, 75% of *TP53* mutated and 68% of CD3^−^CD8 TILs low density cases compared with the best prognosis cluster 1 with 28% of *KRAS* mutated, 52% of *TP53* mutated and 44% of CD3^−^CD8 TILs low density cases. It is known that *KRAS*, *BRAF*, and *MAPK* related mutations decrease the expression of the Major Histocompatibility Complex (MHC) class I genes, as well as the expression of other genes that encode essential peptides cargo molecules. These alterations can reduce the inflammation of tumors and the immunogenic death of their cells by decreasing the density of the ligands available for recognition by T lymphocytes (68, 69). Previous studies have shown that *KRAS* mutation is associated with reduced expression of genes related with innate and adaptive immunity and explicitly suppressing the Th1/cytotoxic immune infiltration in colorectal cancer (31, 70, 71). Recently our group reported that good responders to nCRT displayed a higher density of B cells and were not enriched by KRAS mutations (31). In addition, *TP53* mutations could also lead to immunosuppression processes avoiding the production of crucial chemokines involved in the recruitment of natural killer (NK) cells and T lymphocytes to the tumor microenvironment (72). Consistently, RC cluster 2 tumors showed elevated NPS compared with cluster 1 tumors. NPS is a systemic inflammatory response marker that was significantly associated with a worse CRT response (**Supplementary Table 2**) and has been described in different CRC series independently of TNM, although it has not yet been prospectively validated (73, 74). It is noteworthy that the higher relative values of circulating neutrophils in poor responders (reflected by a higher NPS) and its association with poor survival have been related to neutrophils’ capability to remodel the tumor microenvironment towards a more favorable immunoresistant profile (75).

This real-world setting prospective study of RC patients subject to standard nCRT showed that high NPS and *KRAS* mutated cases are independent predictive factors significantly associated with a worse response to treatment. These results are congruent with previous studies, although these predictive markers are not included in the current guidelines. In addition, we also outlined that mucinous and dMMR RC showed poor tumoral regression after nCRT. Moreover, it was not statistical significative probably due to the low frequency of this features, is clinically significative, reinforcing results of other series. Our analyses also show that high CEA levels and *KRAS* mutational status are independent prognostic factors that could help anticipate worse outcomes during follow-up.

It is important to mention that diverse comorbidities and socioeconomic factors intrinsic with our public health system affected the final sample size of this prospective study due to interruption of the assigned treatments and/or loss of follow-up. In particular, adherence to neoadjuvant treatment was interfered by economic needed that implied the discontinuation of the patient´s treatment, undermining the statistical power of our study.

In conclusion, the comprehensive clinicopathological and molecular characterization of the non-metastatic RC cohort allowed us to identify the most relevant changes and prognostic/predictive factors. More importantly, our findings indicate that two distinctive RC patient clusters with prognostic value can be identified in a multivariate integrative approach, highlighting the synergic role of *KRAS* and *TP53* mutational status with the tumor immune infiltrate. The identified clusters and their associated clinicopathological and molecular factors constitute a framework to develop a risk scoring system that may help to stratify patients with non-metastatic RC at the time of the therapeutic approach. Further independent validation analyses of non-metastatic RC cases need to be performed to evaluate the applicability of our model in the clinical setting.

## Data Availability Statement

The datasets presented in this study can be found in online repositories, and in the **Supplementary Material**.

## Ethics Statement

The studies involving human participants were reviewed and approved by the Udaondo Hospital Ethics Committee (Project Identification code HBU-ONCO-DEGENS, approved May 18, 2015) and the Instituto Leloir Institutional Review Board CBFIL (Project Identification code CBFIL#20, approved May 30, 2015). The patients/participants provided their written informed consent to participate in this study.

## Author Contributions

Conceptualization, SI, MCa, MG, JR, JS, and AL. Methodology, SI, JS, JR, MCa, MK, VM, MR, GR, RS, UG, GM, MAn, MCo, CR, JV, ME, AS, OP, ER, AL, MG., and MAb. Software, validation and formal analysis, SI, MAb, MG, and JR. Investigation and data curation, SI, MAb, MG, JR, JS, and AL. Visualization, SI, MAb, MG, and JR. Writing—Original draft preparation, SI, MAb, MG, and JR. Writing—Review and editing, SI, JS, JR, MCo, MK, VM, MR, GR, RS, UG, GM, MAn, MCo, CR, JV, ME, AS, OP, ER, AL, MG, and MCa. Funding acquisition, SI, MCa, AL, OP, MG, and ER. All authors contributed to the article and approved the submitted version.

## Funding

This research was funded by the Fondation Nelia and Amadeo Barletta (FNAB) and the FS-PBIT 015/13 grant from the FONARSEC, the National Agency for Promotion of Science and Technology, the Ministry of Science, Technology and Productive Innovation, Argentina and the National Council for Scientific and Technological Research (CONICET), Argentina.

## Conflict of Interest

The authors declare that the research was conducted in the absence of any commercial or financial relationships that could be construed as a potential conflict of interest.

## Publisher’s Note

All claims expressed in this article are solely those of the authors and do not necessarily represent those of their affiliated organizations, or those of the publisher, the editors and the reviewers. Any product that may be evaluated in this article, or claim that may be made by its manufacturer, is not guaranteed or endorsed by the publisher.

## Supplementary Material

Supplementary Figure 1 Survival analysis of the significantly associated markers (*KRAS* mutational status and pretreatment CEA levels) with the follow-up of the 76 non-metastatic RC patients as showed by their hazard ratios determined in the univariate (HR) and multivariate (HR*) models.
