# Supplementary figures and images for: Prognostic Impact of An Integrative Landscape of Clinical, Immune, and Molecular Features in Non-Metastatic Rectal Cancer
Source: Front Oncol. 2022 Jan 7;11:801880. doi: 10.3389/fonc.2021.801880 (PMC8777220; doi:10.3389/fonc.2021.801880)

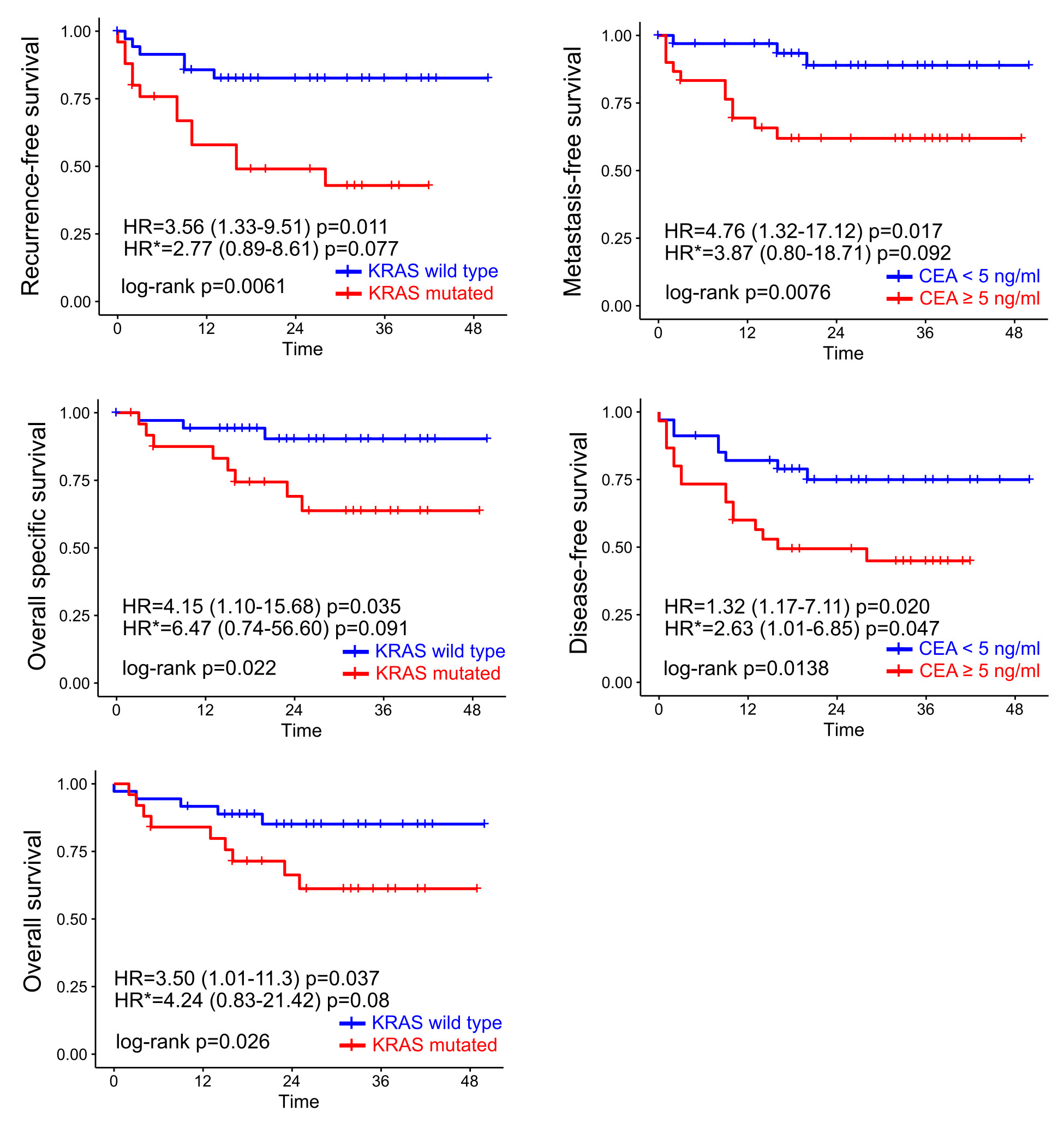

Supplement: Supplementary Figure 1 — Survival analysis of the significantly associated markers (KRAS mutational status and pretreatment CEA levels) with the follow-up of the 76 non-metastatic RC patients as showed by their hazard ratios determined in the univariate (HR) and multivariate (HR*) models. [file Image_1.jpeg]
