# Supplementary material for: Prognostic Impact of An Integrative Landscape of Clinical, Immune, and Molecular Features in Non-Metastatic Rectal Cancer
Source: Front Oncol. 2022 Jan 7;11:801880. doi: 10.3389/fonc.2021.801880 (PMC8777220; doi:10.3389/fonc.2021.801880)
Supplement: Supplementary file 2 [file Table_1.docx]

Supplementary Material

**Supplementary Table 1.** Clinicopathological and demographics data of the non-metastatic rectal cancer cohort according to the assigned treatment.

| **Patient Characteristics ***  (n=76) | **CRT**  (n=25) | **I+CRT**  (n=36) | **Unfront surg.**  (n=15) | **p-value** |
| --- | --- | --- | --- | --- |
| Median age at diagnosis | 63 (54-69) | 59 (45.5-64) | 64 (51-68) | *0.107* |
| Age  ≤ 50 years old  > 50 years old | 5 (20)  20 (80) | 14 (39)  22 (61) | 3 (20)  12 (80) | *0.193* |
| Gender  Female  Male | 8 (32)  17 (68) | 11 (31)  25 (69) | 6 (40)  9 (60) | *0.802* |
| Distance from the anal verge  0-70 mm  71-120 mm  >120 mm | 11 (44)  9 (36)  5 (20) | 17 (47)  15 (42)  4 (11) | 4 (27)  9 (60)  2 (13) | *0.524* |
| NM (RNM)  Stage I (T1-T2, N0)  Stage II (T3-T4, N0)  Stage III (any T, N+). | 1 (4)  18 (72)  6 (24) | 0 (0)  5 (14)  31 (86) | 10 (67)  4 (27)  1 (6) | ***<0.001*** |
| EMVI (RNM)  Positive  Negative | 6 (24)  19 (76) | 18 (50)  18 (50) | 1 (7)  14 (93) | ***0.006*** |
| CRM (RNM)  Positive  Negative | 18 (72)  7 (28) | 34 (94)  2 (6) | 2 (13)  13 (87) | ***<0.001*** |
| Lateral lymph nodes (RNM)  Present  Absent | 1 (4)  24 (96) | 12 (33)  24 (67) | 0 (0)  15 (100) | ***0.001*** |
| CEA (ng/mL)  ≥ 5  < 5 | 12 (48)  13 (52) | 21 (58)  15 (42) | 3 (20)  12 (80) | ***0.044*** |
| CA19.9 (ng/mL)  ≥ 35  < 35 | 6 (24)  19 (76) | 11 (31)  25 (69) | 1 (7)  14 (93) | *0.175* |
| NPS score  1-2  0 | 6 (24)  19 (76) | 14 (39)  22 (61) | 3 (20)  12 (80) | *0.289* |
| Histology  Mucinous  Others | 0 (0)  25 (100) | 7 (19)  29 (81) | 1 (7)  14 (93) | ***0.040*** |
| MMR status  Deficit  Non-deficit | 0 (0)  25 (100) | 4 (11)  32 (89) | 1 (7)  14 (93) | *0.209* |
| CDX2 expression  Negative  Positive | 2 (8)  23 (92) | 0 (0)  36 (100) | 1 (7)  14 (93) | *0.194* |
| HER2/Neu expression  Positive  Negative  Unknown | 1 (4)  23 (96)  1 | 0 (0)  34 (100)  2 | 1 (7)  13 (93)  1 | *0.275* |
| CD3-CD8 TILs density  Low  High  Unknown | 11 (58)  8 (42)  6 | 15 (54)  13 (46)  8 | 5 (50)  5 (50)  5 | *0.914* |
| PD-L1 expression  Negative  Positive  Unknown | 16 (89)  2 (11)  7 | 25 (76)  8 (24)  3 | -  -  - | *0.259* |
| Perineural invasion  Present  Absent  Unknown | 3 (15)  17 (85)  5 | 7 (30)  16 (70)  13 | 2 (13)  13 (87)  0 | *0.393* |
| Vascular invasion  Present  Absent  Unknown | 1 (5)  19 (95)  5 | 8 (35)  15 (65)  13 | 4 (27)  11 (73)  0 | ***0.048*** |
| pEMVI  Present  Absent  Unknown | 1 (5)  19 (95)  5 | 3 (14)  18 (86)  15 | 1 (9)  10 (91)  4 | *0.831* |
| pCRM  Present  Absent  Unknown | 0 (0)  20 (100)  5 | 5 (23)  17 (77)  14 | 0 (0)  15 (100)  0 | ***0.011*** |

* Number of patients (%) unless otherwise stated.
