# Supplementary material for: Prognostic Impact of An Integrative Landscape of Clinical, Immune, and Molecular Features in Non-Metastatic Rectal Cancer
Source: Front Oncol. 2022 Jan 7;11:801880. doi: 10.3389/fonc.2021.801880 (PMC8777220; doi:10.3389/fonc.2021.801880)
Supplement: Supplementary file 3 [file Table_2.docx]

Supplementary Material

**Supplementary Table 2.** Clinicopathological and molecular data of the non-metastatic rectal cancer patients subject to nCRT accordig to CAP groups.

| **Patient Characteristics *** | **CAP 0-1** | **CAP 2-3** | **p-value** |
| --- | --- | --- | --- |
| Median age at diagnosis | 61 (55.5-68) | 59.5 (47-66) | *0.271* |
| Age  ≤ 50 years old  > 50 years old | 4 (20)  16 (80) | 10 (33.3)  20 (66.7) | *0.304* |
| Gender  Female  Male | 5 (25)  15 (75) | 10 (33.3)  20 (66.7) | *0.529* |
| Histology  Mucinous  Others | 1 (5)  19 (95) | 4 (13.3)  26 (86.7) | *0.306* |
| MMR status  dMMR  pMMR | 0 (0)  20 (100) | 4 (13.3)  26 (86.7) | *0.140* |
| CDX2 expression  Negative  Positive | 1 (5)  19 (95) | 1 (3.3)  29 (96.7) | *0.645* |
| HER2/Neu expression  Positive  Negative  Unknown | 0 (0)  20 (100)  - | 1 (3.7)  26 (96.3)  3 | *0.574* |
| Distance from the anal verge  0-70 mm  71-120 mm  >120 mm | 10 (50)  8 (40)  2 (10) | 14 (46.7)  10 (33.3)  6 (20) | *0.687* |
| TNM  Stage I (T1-T2, N0)  Stage II (T3-T4, N0)  Stage III (any T, N+) | 0 (0)  10 (50)  10 (50) | 1 (3.3)  12 (40)  17 (56.7) | *0.744* |
| Mesorectal nodes  Positive  Negative | 10 (50)  10 (50) | 17 (56.7)  13 (43.3) | *0.643* |
| EMVI  Positive  Negative | 7 (35)  13 (65) | 11 (36.7)  19 (63.3) | *0.904* |
| CRM  Positive  Negative | 17 (85)  3 (15) | 24 (80)  6 (20) | *0.652* |
| Lateral lymph nodes  Present  Absent | 2 (10)  18 (90) | 8 (26.7)  22 (73.3) | *0.149* |
| CEA (ng/mL)  ≥ 5  < 5 | 10 (50)  10 (50) | 18 (60)  12 (40) | *0.485* |
| CA 19.9 (ng/mL)  ≥ 35  < 35 | 3 (15)  17 (85) | 10 (33.3)  20 (66.7) | *0.148* |
| NPS score  1-2  0 | 2 (10)  18 (90) | 12 (40)  18 (60) | ***0.021*** |
| CD3  0-34  ≥ 34  Unknown | 11 (78.6)  3 (21.4)  6 | 19 (67.9)  9 (32.1)  2 | *0.469* |
| CD8  0-34  ≥ 34  Unknown | 14 (100)  0 (0)  6 | 27 (96.4)  1 (3.6)  2 | *0.668* |
| PD-L1 expression  Positive  Negative  Unknown | 2 (14.3)  12 (85.7)  6 | 4 (14.3)  24 (85.7)  2 | *0.666* |
| KRAS  Mutated  Non-mutated | 4 (20)  16 (80) | 17 (56.7)  13 (43.3) | ***0.010*** |
| TP53  Mutated  Non-mutated  Unknown | 7 (43.7)  9 (56.3)  4 | 18 (72)  7 (28)  5 | *0.070* |
| CD3-CD8 TILs density  Low  Moderate/High  Unknown | 10 (52.6)  9 (47.4)  1 | 12 (52.2)  11 (17.8)  7 | *0.976* |
| nCRT  I+CRT  CRT | 8 (40)  12 (60) | 19 (63.3)  11 (36.7) | *0.105* |

* Number of patients (%) unless otherwise stated.
