# Supplementary material for: Prognostic Impact of An Integrative Landscape of Clinical, Immune, and Molecular Features in Non-Metastatic Rectal Cancer
Source: Front Oncol. 2022 Jan 7;11:801880. doi: 10.3389/fonc.2021.801880 (PMC8777220; doi:10.3389/fonc.2021.801880)
Supplement: Supplementary file 4 [file Table_3.docx]

Supplementary Material

**Supplementary Table 3.** Univariate and multivariate analysis of the neutrophils-platelets score (NPS) and *KRAS* mutational status as predictive factors of nCRT response based on the CAP groups.

| **Variables** | **Univariate** | | **Multivariate** | | |
| --- | --- | --- | --- | --- | --- |
|  | **OR (IC95%)** | **p-value** | | **OR (IC95%)** | **p-value** |
| Age (≤50 vs. >50 years old) | 2 (0.52-7.58) | 0.308 | | 3.17 (0.52-19.13) | 0.207 |
| Gender (female vs. male) | 1.5 (0.42-5.31) | 0.530 | | 1.48 (0.23-9.25) | 0.670 |
| nCRT (CRT vs. I+CRT) | 2.59 (0.81-8.28) | 0.109 | | 4.48 (0.71-28.14) | 0.109 |
| TNM (Stage 4 vs. 2-3) | 1.30 (0.42-4.07) | 0.643 | | 0.6 (0.10-3.33) | 0.560 |
| *KRAS* (mutated vs. non-mutated) | 5.23 (1.41-19.4) | 0.013 | | 5.49 (1.06-28.40) | 0.042 |
| NPS (1-2 vs. 0) | 6 (1.17-30.72) | 0.032 | | 10.52 (1.34-82.64) | 0.025 |
